# Unveiling Temporal Nonlinear Structure–Rheology Relationships under Dynamic Shearing

**DOI:** 10.3390/polym11071189

**Published:** 2019-07-16

**Authors:** Johnny Ching-Wei Lee, Lionel Porcar, Simon A. Rogers

**Affiliations:** 1 Department of Chemical and Biomolecular Engineering, University of Illinois at Urbana-Champaign, Urbana, IL 61801, USA; 2 Institut Laue-Langevin, B.P. 156, F-38042 Grenoble CEDEX 9, France

**Keywords:** nonlinear rheology, rheo SANS, LAOS, polymer rheology, viscoelastic, worm-like micelles, structure–rheology relationship

## Abstract

Understanding how microscopic rearrangements manifest in macroscopic flow responses is one of the central goals of nonlinear rheological studies. Using the sequence-of-physical-processes framework, we present a natural 3D structure–rheology space that temporally correlates the structural and nonlinear viscoelastic parameters. Exploiting the rheo-small-angle neutron scattering (rheo-SANS) techniques, we demonstrate the use of the framework with a model system of polymer-like micelles (PLMs), where we unveil a sequence of microscopic events that micelles experience under dynamic shearing across a range of frequencies. The least-aligned state of the PLMs is observed to migrate from the total strain extreme toward zero strain with increasing frequency. Our proposed 3D space is generic, and can be equally applied to other soft materials under any sort of deformation, such as startup shear or uniaxial extension. This work therefore provides a natural approach for researchers to study complex out-of-equilibrium structure–rheology relationships of soft materials.

## 1. Introduction

Polymeric materials are ubiquitous in modern society. They are used in environmental [1], biological [2], automotive [3], agricultural [4] and 3D-printing [5] applications, among others. Their success in each particular application is due to their remarkable mechanical properties, which are determined by their molecular-level architecture. The processing conditions that lead to product creation, or the polymer’s end-use, often involve strong or rapidly-changing flow conditions such that the mechanical behaviors change drastically from their intended quiescent states. During these highly out-of-equilibrium intervals, the microstructure potentially undergoes orientational and conformational changes, resulting in complex nonlinear rheological flow behaviors. It is therefore critical to understand how macroscopic responses are manifested from their microscopic or molecular changes under dynamic flow conditions.

Sinusoidal oscillatory shearing is often employed to reliably replicate nonlinear flow conditions, as well as to interrogate material properties [6,7,8,9]. In particular, large-amplitude oscillatory shear (LAOS) offers an ideal experimental protocol to replicate, in a controlled manner, the strong and rapidly-changing flow conditions that are encountered in practical applications, via independent control of length and time scales [10,11,12,13].

In exploring microstructural change under dynamic shearing, several studies have employed distinct imaging or scattering techniques. Boukany and Wang observed that rupture of entangled micelles above a certain critical strain amplitude of LAOS via particle-tracking velocimetry (PTV) [14]. López–Barrón and coworkers observed cyclic melting and recrystallization of a micellar crystal under LAOS via time-resolved small-angle neutron scattering (SANS) [12]. Smith and coworkers [15] exploited confocal microscopy to study particle re-arrangements within colloidal gels under LAOS. Lettinga and coworkers studied nematic platelet dispersions under LAOS via time-resolved small-angle X-ray scattering (SAXS) and reported tumbling motions and a yielding transition [11]. Exploiting X-ray photon correlation spectroscopy (XPCS), and a punctuated LAOS protocol, Rogers and coworkers [16] identified irreversible particle rearrangements of a concentrated colloidal gel. Simulating soft glassy materials, Park and Rogers revealed rich structural dynamics during LAOS, by identifying connections between the changes in the local strain distribution and the macroscopic rheology [17]. These studies, along with many others [18,19,20,21], have exploited advanced imaging or scattering techniques, and have collectively showed that the structure of soft materials under LAOS evolves in a temporal manner, involving physical events that occur sequentially within one period of oscillation.

At the macroscopic level, previous attempts to understand LAOS rheology typically *average* responses over a cycle of deformation and therefore extract average parameters of sorts. The most common of these is Fourier transformation of time-domain stress responses to determine the spectrum of Fourier harmonics [22]. Other Fourier-based frameworks include the stress decomposition [23] and the Chebyshev description of the stress decomposition parameters [24]. Despite being mathematically robust, these approaches lack clear physical meaning, which has discouraged researchers from trying to correlate the Fourier metrics to the underlying structural measurements either from confocal microscopy [21], simulation [17], or scattering techniques [25]. In addition, the assumptions that are central to the stress decomposition scheme, on which the Chebyshev description is based, have been shown not to be generally true by experimental, theoretical, and simulation studies [17,26,27,28]. Specifically, it has been demonstrated that microstructure of polymeric materials evolves as a sequence of physical events under LAOS, and the sequences undergone do not display the symmetries assumed by the stress decomposition. Performing stress decomposition or its derivative analyses therefore averages macroscopic responses in physically and structurally different stages, and results in parameters that conflate rheologically distinct states. Because these analysis methods average macroscopic responses over a cycle of deformation, structural rearrangements that occur with time scales shorter than a full period of oscillation [11,12,14,15,16,18,19,20,21,28] are difficult, or impossible, to identify.

Macroscopic rheological responses and microscopic structure are causally related. The goal of time-resolved hybrid rheo-X experiments, where X could be SANS [25], SAXS [29], NMR [30,31], XPCS [18], small-angle light scattering (SALS) [32], confocal microscopy [33,34], PTV [14] etc., is the formation of so-called structure–rheology relationships. Macroscopically, oscillatory-shear responses are often decomposed into parameters that gauge elastic and viscous properties. This viewpoint takes its cues from the use of the dynamic moduli, $G^{\prime }$ and $G^{\prime \prime }$, in the linear-viscoelastic regime, where the dynamic moduli represent the average amount of energy storage and loss per unit volume over a cycle of deformation [35]. Equivalently, microscopic processes can be attributed to elastic or viscous viewpoints, or sometimes their combination. For instance, polymeric chains that are aligned in a flow field result in the shear-thinning behavior exhibited by most synthetic polymers. This correlation between molecular alignment and shear-thinning clearly takes a viscous perspective. Conversely, stretching of biopolymer networks that produces strain stiffening due to entropic or geometrical changes [36,37] is a microscopic process that leads to a macroscopically elastic response. While such structure–rheology links have been clearly formed in nearly purely viscous and purely elastic cases, a framework that temporally correlates structure and rheology from a more general (nonlinear) viscoelastic perspective is needed.

**Figure 1:**
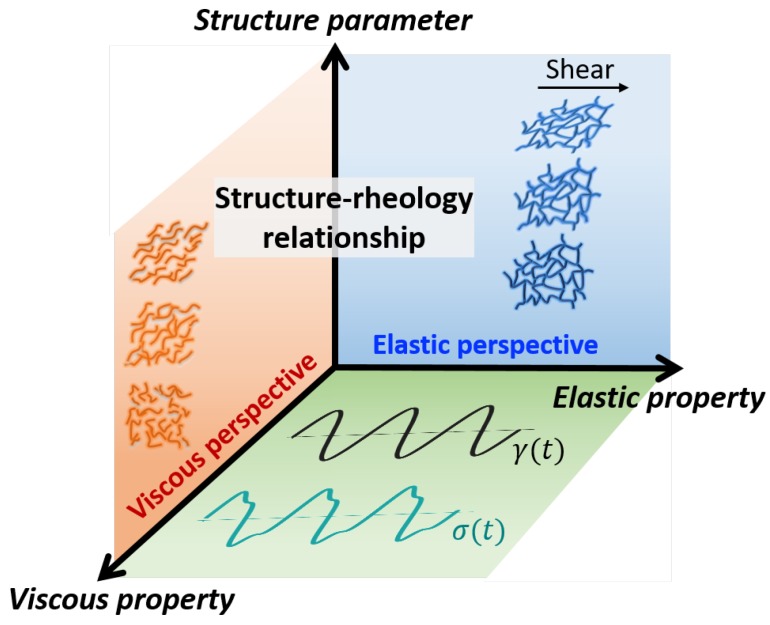
A 3D-schematic of structure–rheology relationships.

We propose that simple structure–rheology relationships exist within a generic 3D space defined by the apparent time-resolved elastic and viscous properties as well as a time-resolved structure parameter, as illustrated in Figure 1. This structure–rheology space allows researchers to not only temporally link macroscopic responses to their microscopic causes, but also allows for the identification of these processes in a clearly elastic or viscous perspective. Formation of trajectories in this space hence facilitates the understanding of complex structure–rheology relationships. At the moment, we leave the parameters of the space generic. Specific choices ought to be driven by the needs of a particular application, material, or process. For instance, numerous structure parameters have been used in the study of oscillatory shearing, including, but not limited to: microscopic yielding rate [17]; alignment factor [20,28,38]; order parameter [11,26,39,40]; degree of order [12]; degree of banding [41]; fractional extension [21]; and nematic order parameter [42]. Within our proposed framework, the structural measure could be any one of these parameters, or another measure not listed here.

To fulfill the need to continuously monitor elastic and viscous parameters with complex LAOS stress responses that would constitute the bottom plane of Figure 1, we make the specific choice to use the sequence of physical processes (SPP) framework, which determines the instantaneous elastic and viscous properties moduli, $G_{t}^{\prime }$ and $G_{t}^{\prime \prime }$ [43,44]. These parameters have been shown to address the symmetry issues that effect the stress decomposition, Chebyshev, and Fourier approaches. Park and Rogers have recently shown that these parameters also clearly identify structural changes in soft glassy materials [17], while Donley and co-workers have successfully identified yielding transitions using SPP metrics [45]. Beyond the conventional dynamic moduli in the linear regime, the instantaneous elastic and viscous moduli of the SPP approach are well-defined in the complex nonlinear regime and are not restricted to oscillatory shearing protocols. By using the SPP analysis, the structure–rheology space is therefore generically applicable to other sophisticated protocols such as parallel superposition [46].

We exploit time-resolved rheo-SANS techniques [25] to study a polymer-like micelle (PLM) solution. Above a critical concentration, the PLMs spontaneously form an entangled network that behaves like an entangled uniform polymer solution [47]. In addition to the typical reptation dynamics of entangled polymers [48], PLMs constantly break and reform on a time scale shorter than their reptation time, leading to Maxwellian behaviors in the linear regime [47]. Owing to their unique dynamics, they have therefore been extensively used to study entangled linear and branched polymers [14,39,49,50,51]. While the linear viscoelasticity of PLMs has been well studied experimentally and theoretically [47,52], their nonlinear dynamics and structural correlation under LAOS remain an active area of research [26,28,38,49]. Combining with structural information provided from in-situ SANS, we demonstrate, using LAOS as a model nonlinear flow protocol, how the rich underlying structure–rheology relationships can be unveiled with the proposed 3D space.

## 2. Materials and Methods

### 2.1. Materials

We studied a polymer-like micellar solution consisting of cetylpyridinium chloride (CPCl) and sodium salicylate (NaSal) in D${}_{2}$O. The polymeric solution had 3.58 wt % CPCl with the molar ratio of NaSal to CPCl of 0.65, and was prepared at least two days before experiments to allow for equilibration. The micellar solution is investigated at two different temperatures, 23.5 and 30 ${}^{\circ }$C to provide access to different frequency regimes.

### 2.2. Rheo-Small-Angle Neutron Scattering

**Figure 2:**
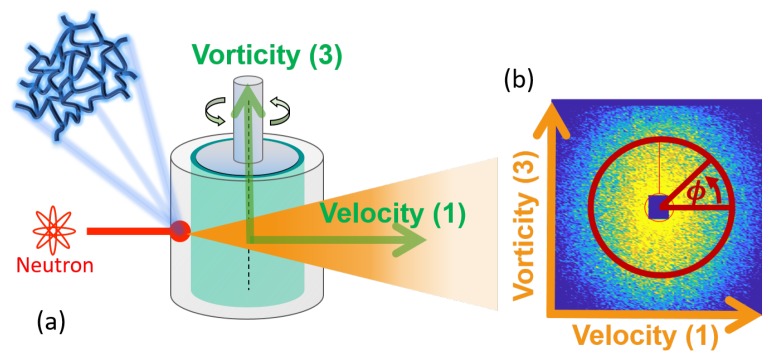
A schematic of the rheo-small-angle neutron scattering (rheo-SANS) experiment setup. (**a**) A quartz couette geometry is used to impose dynamic flow fields. Microstructure is continuously monitored throughout the experiments, as temporally captured by neutron detector, forming the 2D SANS pattern (**b**).

Small-angle neutron scattering experiments were conducted at the Institut Laue–Langevin (Grenoble, France) on the D22 SANS beamline. An MCR501 rheometer (Anton–Paar, Graz, Austria), equipped with a concentric cylinder Couette quartz geometry with a 1 mm gap ($R_{1}=24$ mm; $R_{2}=25$ mm) was used for all experiments. Structure in the 1–3 (flow-vorticity) plane was continuously measured, as shown in Figure 2. We configured the SANS with a neutron wavelength of 6 Å and a detector distance of 11 m, resulting in a *q*-vector range corresponding to Kuhn segments of micelles, 0.006 1/Å to 0.03 1/Å.

Time-resolved SANS methods [25] were used to continuously monitor the microstructural change of micelles under steady-alternating LAOS states, and the 2D SANS data are reduced using LAMP and ILL GRASP (Institut Laue-Langevin (ILL), Grenoble, France) with a temporal deconvolution protocol [53] to enhance the resolution and accuracy.

To quantify the shear-induced orientation of micellar Kuhn segments, the resulting 2D scattering patterns (Figure 2b) were reduced to a single scalar, $A_{f}$,

(1) $$A_{f}=\int_{0}^{2\pi }I_{c}\left(q^{*},\phi \right)cos\left(2\left(\phi -\phi_{0}\right)\right)d\phi /\int_{0}^{2\pi }I_{c}\left(q^{*}\right)d\phi ,$$

where $I_{c}\left(q^{*},\phi \right)$ is the azimuthal intensity over $q^{*}$, and ϕ is the azimuthal angle with the Kuhn segmental q-range $q^{*}=0.006$ to 0.03 1/Å. $\phi_{0}$ represents an orientation angle. The alignment factor, $A_{f}$, will act as the structural measure in our 3D space.

### 2.3. Sequence of Physical Processes (SPP)

The sequence of physical processes framework [43] was used to analyze the raw LAOS waveform. An open-source MATLAB-based software [44] was used to determine the instantaneous elastic and viscous moduli, $G_{t}^{\prime }\left(t\right)$ and $G_{t}^{\prime \prime }\left(t\right)$, during each LAOS cycle. In addition to providing instantaneous elastic and viscous moduli, the SPP framework also allows researchers to estimate the amount of recoverable strain in the system at instant when $G_{t}^{\prime }$ is larger than $G_{t}^{\prime \prime }$. This capability is unique among all methods for analyzing LAOS responses. All the LAOS analysis was performed with data from the steady alternating state. An in-depth discussion of the SPP framework can be found elsewhere [17,43,44].

## 3. Results

### 3.1. Linear Viscoelasticity

**Figure 3:**
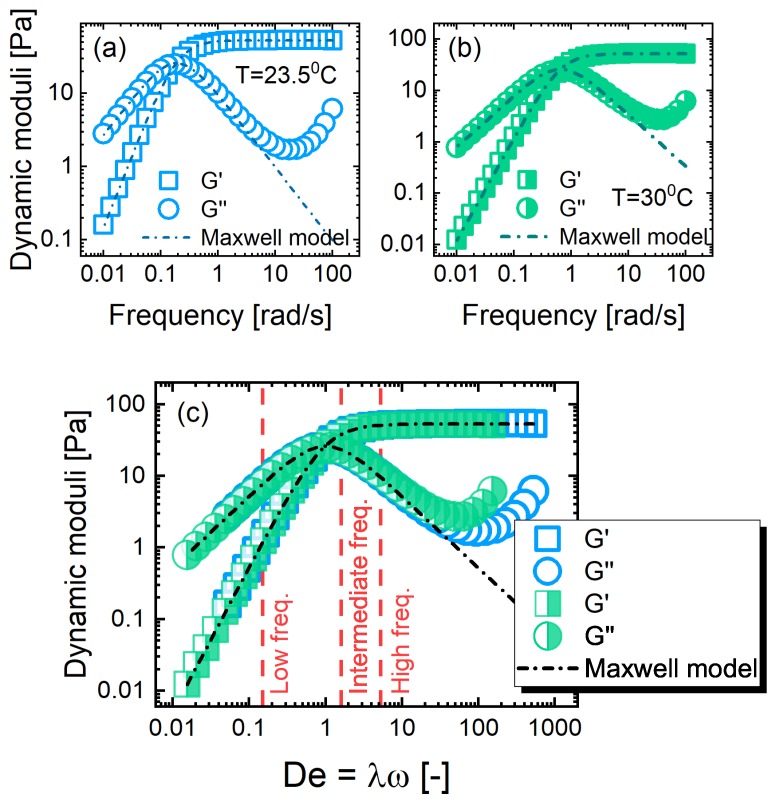
Linear viscoelastic responses. The frequency dependence of dynamic moduli $G^{\prime }\left(\omega \right)$ and $G^{\prime \prime }\left(\omega \right)$ from the polymer-like micelles (PLMs) at 23.5 ${}^{\circ }$C (**a**) and 30 ${}^{\circ }$C (**b**), where the dashed line represents a fit from single-mode Maxwell model. (**c**) The dynamic moduli are collectively plotted against their normalized frequencies De=λω. The three distinct investigated LAOS frequencies are labeled, corresponding to the terminal, rubbery-to-terminal and rubbery–plateau dynamics.

We begin by presenting the linear viscoelastic behavior of the PLM solutions. The frequency-dependence of the dynamic moduli, $G^{\prime }\left(\omega \right)$ and $G^{\prime \prime }\left(\omega \right)$, is displayed in Figure 3 at the two investigated temperatures. A fit from a single-mode Maxwell model is provided as dashed lines. The linear-regime behavior of the PLM solutions was Maxwellian, as laid out previously, due to their reptation, breakage, and reformation dynamics.

We present the responses as a function of the Deborah number, which was a normalized frequency (De=λω, where ω is the imposed frequency and λ is the relaxation time determined from the crossover of dynamic moduli) in Figure 3c. The consistency of the dynamics, as determined by this presentation confirms that the PLMs undergo the same processes at the two investigated temperatures. The effect of temperature was therefore to change the dynamical regime, which we exploit to access higher Deborah numbers.

### 3.2. LAOS as a Model Protocol

We use large amplitude oscillatory shear to enforce nonlinear flow conditions on the PLMs with well-defined length and time scales. We vary the imposed frequency to extend the range of studied dynamics, and focus on low (De=0.15), intermediate (De=1.66) and high (De=5.26) frequencies that correspond to the terminal relaxation, crossover, and plateau regimes under linear viscoelastic conditions. The three investigated LAOS frequencies are labeled in Figure 3c.

**Figure A1:**
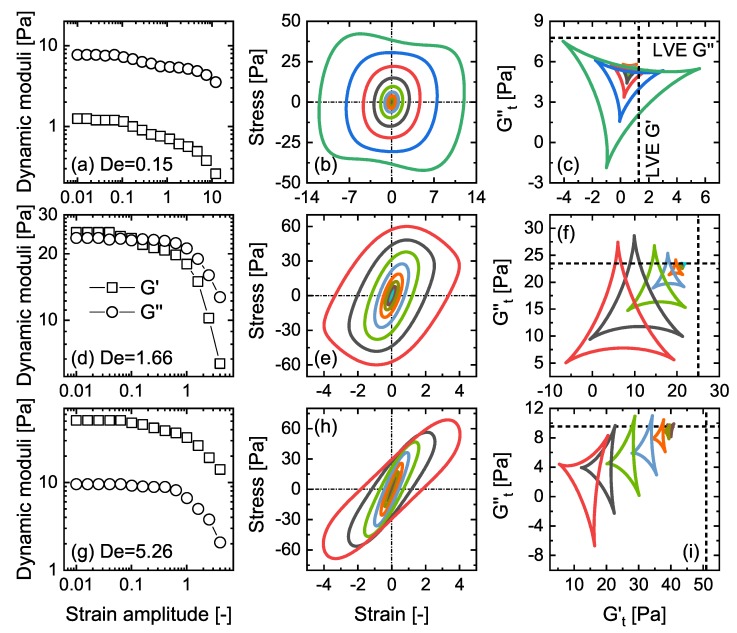
Amplitude dependence of oscillatory shear responses. (**a**,**d**,**g**) Dynamic moduli ($G^{\prime }$, $G^{\prime \prime }$) as a function of applied strain amplitude. (**b**,**e**,**h**) stress versus strain across the amplitudes. (**c**,**f**,**i**) time-dependent viscous modulus versus time-dependent elastic modulus ($G_{t}^{\prime \prime }-G_{t}^{\prime }$) across the amplitudes.

The amplitude dependence from the three distinct frequencies is presented in Figure A1. In an average sense, the transition of dynamic moduli from linear viscoelastic (LVE) to LAOS regimes is shown in Figure A1a,d,g. The oscillatory shear responses across the amplitudes are displayed in Figure A1b,e,h, where the SPP analysis is applied to study their local instantaneous viscoelasticity. The SPP moduli are plotted parametrically against each other in Figure A1c,f,i. Within this presentation, upward, downward, rightward, and leftward motion of the trajectory clearly indicates macroscopic thickening, thinning, stiffening, and softening, respectively [17,43].

**Figure 4:**
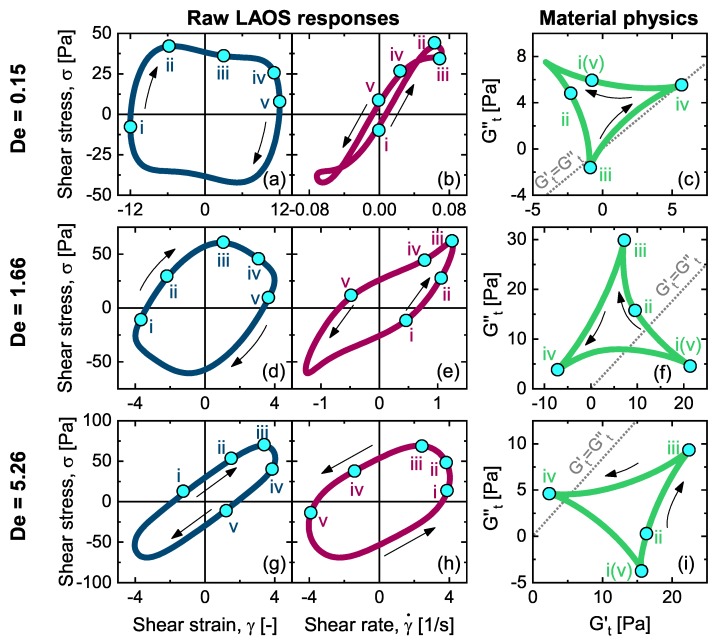
Raw large-amplitude oscillatory shear (LAOS) responses and associated material physics. (**a**,**d**,**g**) Elastic Lissajous curve of shear stress versus shear strain; (**b**,**e**,**h**) Viscous Lissajous curve of shear stress versus shear rate. (**c**,**f**,**i**) Physical interpretation of LAOS responses constituted by plotting time-dependent viscous modulus versus time-dependent elastic modulus.

We focused on three particular LAOS responses at the three distinct frequency regimes as presented in Figure 4 to further study their microstructural origins. The complex evolution of the distorted curves implies that the material was well into the nonlinear regime and experiences significant structural rearrangement within a half cycle of oscillation. The SPP analysis [43,44] was therefore used to elucidate the underlying material physics. We label numerous instants along the oscillatory shear responses to facilitate detailed discussion in the following sections.

### 3.3. Temporal Structure–Rheology Relationships

**Figure 5:**
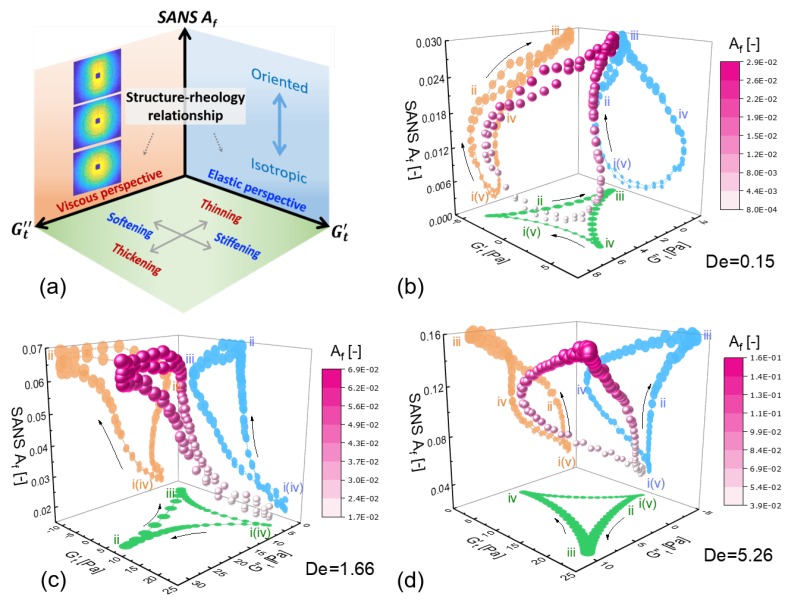
Microstructural evolution is coupled with rheology via rheo-SANS and sequence of physical processes (SPP) techniques under dynamic shearing. (**a**) 3D space $\left[G_{t}^{\prime },\;G_{t}^{\prime \prime },\;A_{f}\right]$ constituted by the time-dependent SPP moduli and SANS alignment factor. The structure–rheology correlation under LAOS laid out by the 3D space of $\left[G_{t}^{\prime },\;G_{t}^{\prime \prime },\;A_{f}\right]$ at the investigated normalized frequencies of De=0.15 (**b**), De=1.66 (**c**) and De=5.26 (**d**), where the degree of alignment is indicated with a color scale.

To study the nonlinear structure–rheology relations, we simultaneously monitored their microstructural evolution and rheological responses under LAOS via SPP-rheo-SANS. We exploited the SPP analysis scheme to quantify elastic ($G_{t}^{\prime }$) and viscous ($G_{t}^{\prime \prime }$) properties, and reduce the 2D SANS pattern into the alignment factor ($A_{f}$). The resulting 3D space was therefore defined by $\left[G_{t}^{\prime },\;G_{t}^{\prime \prime },\;A_{f}\right]$, as shown in Figure 5a. In the bottom plane defined by $G_{t}^{\prime \prime }$-$G_{t}^{\prime }$, the physical meanings, which have been studied previously [17,43], were clear. Motion around the trajectory indicated stiffening, softening, thickening, and thinning. The SANS $A_{f}$ was used to temporally correlate the macroscopic responses to the degree of alignment, where upward and downward motions within a trajectory indicate that segments were being aligned and made more isotropic, respectively. An immediate benefit of the 3D nature of the proposed space was that alignment of Kuhn segments can lead to either elastic or viscous effects in the bulk. By being able to correlate alignment with the instantaneous SPP moduli, we were able to clearly differentiate between alignment of an intact elastic entangled network and alignment of a broken network within a viscously flowing material.

The temporal structure–rheology relationships of PLMs at the three distinct frequencies are displayed in Figure 5b–d, with associated projections in each plane. Notably, the trajectory in the $\left[G_{t}^{\prime },\;G_{t}^{\prime \prime },\;A_{f}\right]$-space traces the same path twice per cycle of oscillation. This was because the SPP instantaneous moduli and the SANS $A_{f}$ were independent of shear direction. The symmetries that were shared by the SPP moduli and the SANS $A_{f}$ suggest that the SPP framework was the natural representation of dynamical structural measurements.

The complexity of the 3D trajectories in Figure 5 implies that the underlying structure–rheology relations are rich with detail. We have labelled a number of points of interest in each trajectory and focus on each regime individually in the following sections.

### 3.4. Low-Frequency Regime (De<1)

The low-frequency dynamics (De=0.15) of PLMs, with a period of oscillation that is longer than the relaxation time ($\omega^{-1}>\lambda$), resemble that of an entangled polymer network. The polymeric micelles have enough time to undergo reptation motion at this frequency, giving rise to re-entanglement and dis-entanglement features [54]. We note that the investigated time scale ($\omega^{-1}=10$ s) is still shorter than the reptation time of the PLMs, where $\lambda_{rep}=\lambda^{2}/\lambda_{br}\approx 70$ s, and λ is the relaxation time determined at the crossover of $G^{\prime }$ and $G^{\prime \prime }$, and $\lambda_{br}$ is the time scale of breakage and recombination [47]. These time scales, however, were approximated from small perturbations where no disentanglement events happen. Whether the system follows the same time scales when displaying the same dynamics (reptation with scission and recombination), when experiencing periodic nonlinear deformation was still unknown.

**Figure 6:**
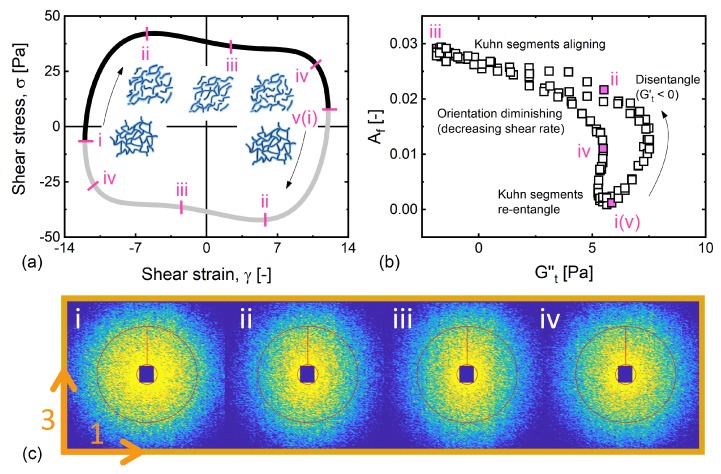
The microstructural evolution during the course of oscillation at De=0.15. The structure evolves in a sequence: network disentangling in stages i–ii, Kuhn segments aligning in stages ii–iii, orientation diminishing with decreasing shear rate in stage iii–iv, and Kuhn segments re-entangling to form the initial quiescent network in stages iv–v. (**a**) Shear stress versus shear strain. (**b**) The degree of alignment is plotted as a function of time-dependent viscous modulus. (**c**) 2D SANS patterns in the corresponding stages during a LAOS cycle.

We present the macroscopic LAOS response (Weissenberg number, $We=\lambda {\dot{\gamma }}_{0}\approx 2$) in Figure 6a as shear stress versus strain in a so-called elastic Lissajous–Bowditch figure. While the elastic projection of our proposed structure–rheology space ($G_{t}^{\prime }-A_{f}$) in Figure 5b displays a circular pattern (indicating weak correlation), the viscous projection ($G_{t}^{\prime \prime }-A_{f}$) shows a strong correlation above a critical alignment. This agrees with the previous findings [26], and can also be seen from the dominance of the viscous modulus over the elastic modulus $G_{t}^{\prime \prime }>G_{t}^{\prime }$ feature in Figure 4c. The viscous projection of the structure–rheology space ($G_{t}^{\prime \prime }-A_{f}$) is therefore displayed in Figure 6b with corresponding 2D SANS patterns in Figure 6c. We label five distinct instants in Figure 6 to facilitate the understanding and discussion.

At stage i, close to the strain extremes, we observed isotropic scattering patterns with negligible alignment that is characteristic of the quiescent structure. The phenomenology that linear viscoelasticity and quiescent structure occur at the strain extreme, has been reported by several groups studying a range of soft materials [43,55,56,57,58,59]. This behavior has recently been rationalized [28] by invoking the concept of recoverable strain. We note that the SPP analysis scheme, unlike all other LAOS analysis schemes, takes the recoverable strain into account by considering a moving reference strain in its construction. However, at this low frequency, the condition required to be able to determine the recoverable strain via the SPP approach ($G_{t}^{\prime }>>G_{t}^{\prime \prime }$) is not met.

During the interval from i to ii, the nearly unchanged viscous modulus ($G_{t}^{\prime \prime }\approx$ 7 Pa) and the decrease of the instantaneous elastic modulus indicate that the system undergoes disentanglement events ($dG_{t}^{\prime }/dt<0$) resulting from network straining. Some segments therefore align. Once the network has fully disentangled by point ii, the increasing shear rate results in more alignment of Kuhn segments, with the state of highest alignment being observed around the shear rate extreme (point iii). This microscopic aligning process from points ii to iii is also manifested in the macroscopic thinning determined by the decrease in $G_{t}^{\prime \prime }$ with an unchanged elastic modulus ($G_{t}^{\prime }$). As the shear rate decreases again between points iii and iv, we observe a diminishing alignment associated with macroscopic thickening ($dG_{t}^{\prime \prime }/dt>0$). The term `thickening’ here requires some clarification. It is not used in the context of shear-thickening, which is identified as an increase in viscosity with an increase in shear rate. Rather, this is a more general descriptor that identifies an increase in viscous dissipation. In this case, the increase in viscosity occurred as the shear rate decreases, which was a natural consequence of decreasing the shear rate in a shear-thinning material. The decrease of alignment and corresponding thickening process follows nearly the same path as the previous aligning/thinning process, with only a slight deviation close to point iv, adding to the simplification of interpretation. This behavior was consistent with a partial re-entanglement as the shear rate approaches zero [54] of a shear-thinning response. In the process that goes from point iv to point v, where the rate goes to zero, the chains re-entangle and reform the initial quiescent network structure, as evidenced by the maximum in $G_{t}^{\prime }$ and negligible alignment at point v. The identical sequence of events was observed again, but in the other direction, before the period of oscillation was completed.

### 3.5. Intermediate-Frequency Regime (De≈1)

At a frequency close to the crossover of the dynamic moduli in the linear regime (De=1.66), but with a much larger amplitude that would elicit linear dynamics (Wi=6.6), we see that the elastic and viscous projections in Figure 5c are both non-trivially correlated to the structural evolution. In this regime, polymer-like micelles have less time to undergo reptation motion than the low-frequency dynamics, and so are more elastically strained.

**Figure 7:**
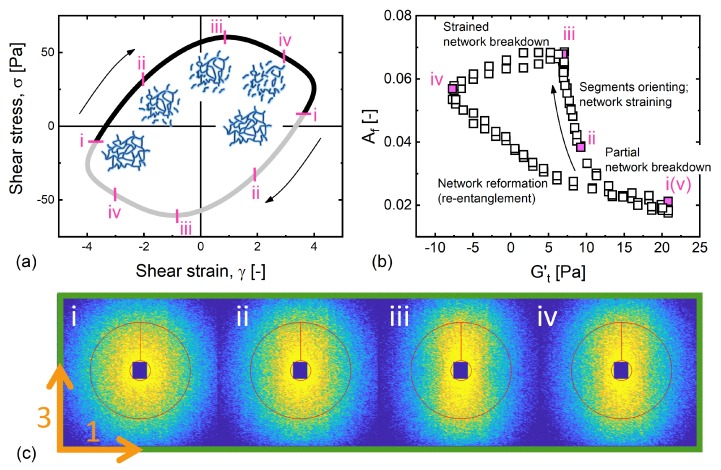
The microstructural evolution during the course of oscillation at De=1.66. The structure evolves in a sequence: network partially disentangling and broken in stages i–ii, Kuhn segments aligning with the rest of network being strained in stages ii–iii, orientation slightly diminishing in stages iii–iv due to decreasing shear rate, and Kuhn segments re-entangling to reform initial network in stages iv–v. (**a**) Shear stress versus shear strain. (**b**) The degree of alignment is plotted as a function of time-dependent elastic modulus. (**c**) 2D SANS patterns in the corresponding stages during a LAOS cycle.

We present the elastic Lissajous curve, the elastic projection of the 3D-space ($G_{t}^{\prime }-A_{f}$) and the respective reduced 2D SANS patterns in Figure 7. Each figure is labeled with numbers at several instants (i–v) to assist the discussion.

Starting at point i, where the instantaneous elastic SPP modulus $G_{t}^{\prime }$ was largest, and the least alignment was measured, we identified a micellar network that was slightly aligned. While a total strain of approximately four was applied, the recoverable strain estimated by the SPP indicates the recoverable strain was only about 0.4 [43,44]. This implies that the material experienced a much smaller strain than the total strain of four and therefore exhibits little alignment. With further straining, the network macroscopically softened as it started to be broken down ($dG_{t}^{\prime }/dt<0$). In the process from point i to ii and on to point iii, freed segments and the strained network were aligned in the flow direction. Immediately after the stress maximum at point iii, the sharp decrease of $G_{t}^{\prime }$ suggests the network was almost completely broken down into segments [17] resulting in the thinning nature ($dG_{t}^{\prime \prime }/dt<0$) at the high shear-rate portion. The alignment slightly declines because of the decreasing shear rate from point iii to point iv. During the last process between points iv and v, we observe a diminishing alignment coupled with a rapidly increasing $G_{t}^{\prime }$, indicating that the entangled network is being reformed. A nearly unchanged $G_{t}^{\prime \prime }$ during this process further suggests that this reformation was a predominantly elastic process. This sequence of events was observed twice per oscillation, both in rheology and structure, constituting the time-resolved motion that traces the same trajectory in Figure 5 twice in a cycle.

### 3.6. High-Frequency Regime (De>1)

In the high-frequency regime (De=5.26, Wi=21), it is known that the system responds to applied deformation in an predominantly elastic manner, as $G_{t}^{\prime }>G_{t}^{\prime \prime }$ can be seen in Figure 4i. Rogers and coworkers have qualitatively observed that in a portion of oscillation at a high frequency, the alignment develops with applied strain [26]. Coupling the SPP and SANS techniques in this section, we quantitatively unveiled the underlying structural and rheological transition throughout the entire course of an oscillation.

**Figure 8:**
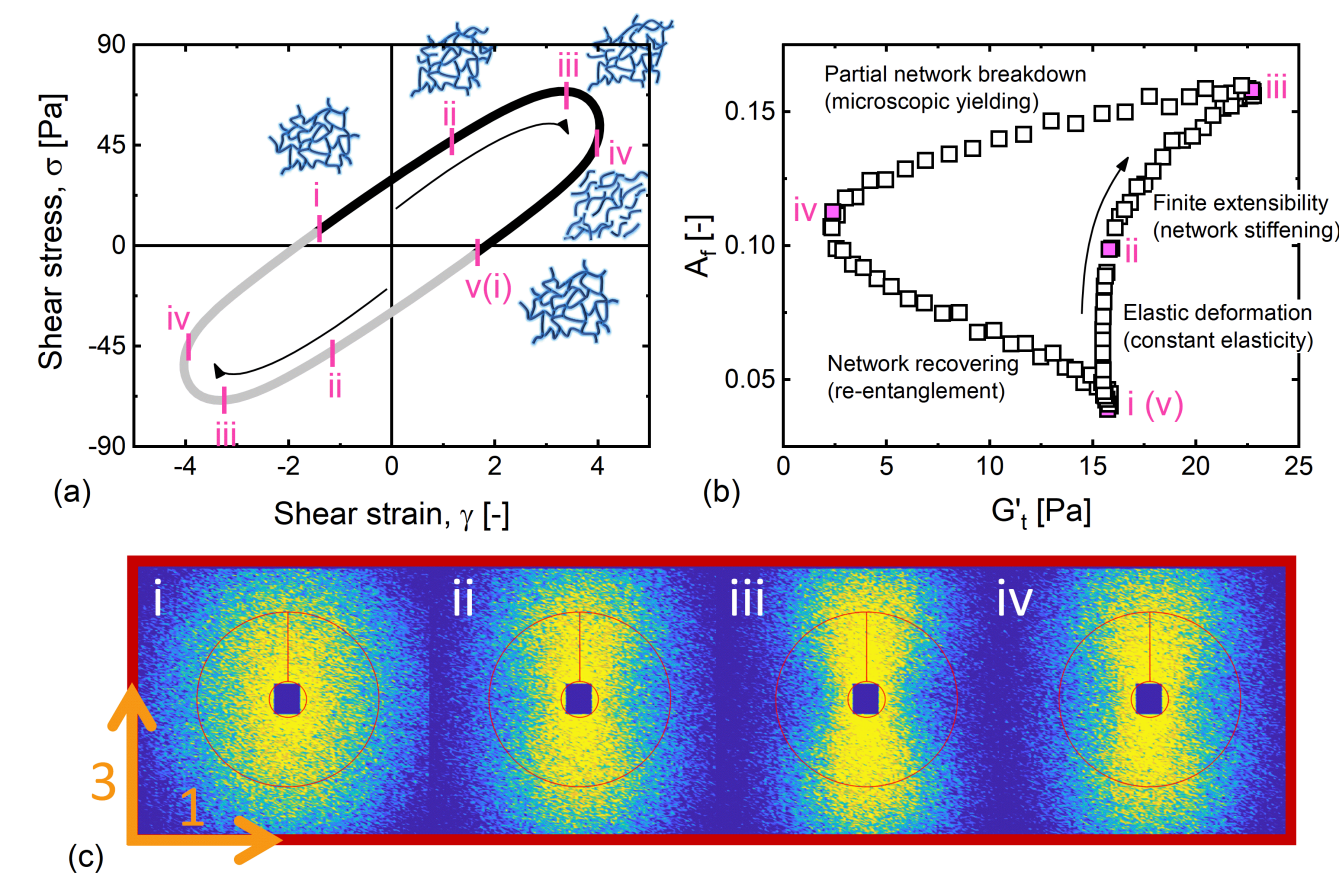
The microstructural evolution during the course of oscillation at De=5.26. The structure evolves in a sequence: network undergoing purely elastic deformation in stages i–ii, further stretching network resulting in stiffening responses (finite extensibility) in stages ii–iii, network broken into segments (microscopic yielding) at large strain in stages iii–iv, and network reforms to initial state in stages iv–v. (**a**) Shear stress versus shear strain. (**b**) The degree of alignment is plotted as a function of time-dependent elastic modulus. (**c**) 2D SANS patterns in the corresponding stages during a LAOS cycle.

The elastic Lissajous curve, the elastic projection of the 3D SPP-rheo-SANS space and respective scattering patterns are presented in Figure 8. In the process that occurs between points i and ii, where a linear correlation between the strain and order parameter was observed [26], we see a constant elastic SPP modulus $G_{t}^{\prime }$ that was much larger than $G_{t}^{\prime \prime }$, indicating that the increasing alignment was due to the entangled network being strained, and that the network undergoes elastically affine deformation, where the recoverable strain increases linearly from −0.81 (i) to 3 (ii) as revealed by the SPP. Above a certain degree of straining around point ii, we observe a macroscopic stiffening response from the network, resulting from the finite extensibility of the network strands [60]. We therefore see that the structural alignment coherently evolved with the (recoverable) strain.

Immediately after the stress maximum was encountered at point iii, we observe a sharp decrease in the elastic SPP modulus toward point iv. The softening indicated by the decrease in $G_{t}^{\prime }$ suggests that the network partially breaks down and transitions into segments that are prone to flow. As the shear rate at this point is close to zero, the alignment also decreases, which in turn allows for the reformation of the entangled network in the process between points iv and v.

### 3.7. Discussion

Combining the results from the three distinct frequency regimes, some features and similarities are noteworthy. The least aligned state, which is close to the equilibrium quiescent structure that we have denoted as point i in all conditions is located at, or close to, the point of zero stress. This point of quiescent structure and low stress migrates from the total strain extreme at the lowest frequency toward the point of zero strain at the highest frequency. In all cases, the SPP-calculated recoverable strain is smaller than the total strain at the same point. That is, the instant of zero recoverable strain, where isotropic scattering is expected, moves gradually toward zero total strain with an increase in frequency.

In addition, across the three frequency regimes, we identify a breaking of the entangled network into segments or shorter unentangled polymer-like micelles at the instant of stress overshoot, such that a macroscopic softening motion ($dG_{t}^{\prime }/dt<0$) is observed. We remark that this phenomenon qualitatively agrees with the findings from entangled polymers upon start-up shear [61] or uniaxial extension [62], that microscopic yielding events occur at the stress-overshoot where complete strain recovery can no longer persist [28,63]. This phenomenon suggests uni-directional, steady shear features can exist in a LAOS protocol that involves shear reversal [28,64].

## 4. Conclusions

Many polymeric materials undergo intervals of rapidly-changing strong flows when being processed, transported, or during their intended application. It is therefore imperative to have a rheological framework that allows a temporal coupling of microscopically structural evolution to macroscopic flow responses. We have presented such a framework here, in the form of a generic 3D space defined by elastic and viscous properties as well as a structural measure. We have made specific choices of which parameters to use in this generic space, and shown that they provide clear and concise understandings of structural and rheological evolution under oscillatory shearing.

Exploiting in-situ SANS techniques and LAOS as a model nonlinear flow protocol, we have demonstrated how structure–rheology relationships can be clearly unveiled by the proposed 3D-structure–rheology space, with an entangled network of polymer-like micelles. We make the specific choice to use the SPP framework to continuously monitor the elastic and viscous macroscopic properties under oscillatory shearing providing two dimensions of the 3D space. The SPP metrics have been shown to be useful in interpreting the structural progression of other soft materials, and trace the identical trajectory twice per oscillation. They therefore constitute the natural space in which to investigate structure–rheology relationships.

Coupling rheological and structural responses, we have clearly identified sequences of microscopic events that the PLMs experience during the course of oscillations at low, high, and intermediate frequencies. Overall, the transient network breaks down after being strained too much, and the material response then transitions into behaviors that are rate-dependent. As the shear rate decreases to zero, the network reforms and regains its initial structure and rheological properties. We have also observed that the least-aligned instant, where the structure is most like the equilibrium state, shifts from being close to the strain extreme at low frequencies to being close to the point of zero strain at high frequencies. This observation is consistent with the conclusion that strain is acquired mostly in an unrecoverable manner at a low frequency and recoverably at a high frequency.

We have presented a structure–rheology coupling method that can be generically applied to other polymeric materials. We have demonstrated the use of the framework to unveil the temporal correlation of a particular polymer-like micellar solution, but the method can be applied to any polymeric or soft material with any sort of deformation protocol, such as startup shear or extensional rheology. This work therefore serves as a rigorous and natural approach for researchers to more deeply study the complex structure–rheology relationships exhibited by polymers under strong and rapid deformation protocols.
